# Assessment of p53 and ATM functionality in chronic lymphocytic leukemia by multiplex ligation-dependent probe amplification

**DOI:** 10.1038/cddis.2015.223

**Published:** 2015-08-06

**Authors:** G D te Raa, P D Moerland, A C Leeksma, I A Derks, H Yigittop, N Laddach, M Loden-van Straaten, V Navrkalova, M Trbusek, D M Luijks, T Zenz, A Skowronska, M Hoogendoorn, T Stankovic, M H van Oers, E Eldering, A P Kater

**Affiliations:** 1 Department of Hematology, Academic Medical Center, Amsterdam, The Netherlands; 2 Laboratory of Experimental Immunology, Academic Medical Center, Amsterdam, The Netherlands; 3 Bioinformatics Laboratory, Department of Clinical Epidemiology, Biostatistics and Bioinformatics, Academic Medical Center, Amsterdam, The Netherlands; 4 MRC-Holland, Amsterdam, The Netherlands; 5 Department of Molecular Medicine, Central European Institute of Technology, Masaryk University, Brno, Czech Republic; 6 Department of Translational Oncology, National Center for Tumor Diseases (NCT), German Cancer Research Center (DKFZ) and Department of Medicine V, University Hospital Heidelberg, Heidelberg, Germany; 7 School of Cancer Sciences, University of Birmingham, Birmingham, UK; 8 Department of Hematology, Medical Center Leeuwarden, Leeuwarden, The Netherlands; 9 LYMMCARE (Lymphoma and Myeloma Center), Amsterdam, The Netherlands

## Abstract

The ATM-p53 DNA-damage response (DDR) pathway has a crucial role in chemoresistance in CLL, as indicated by the adverse prognostic impact of genetic aberrations of *TP53* and *ATM*. Identifying and distinguishing *TP53* and *ATM* functional defects has become relevant as epigenetic and posttranscriptional dysregulation of the ATM/p53 axis is increasingly being recognized as the underlying cause of chemoresistance. Also, specific treatments sensitizing *TP53*- or *ATM*-deficient CLL cells are emerging. We therefore developed a new ATM-p53 functional assay with the aim to (i) identify and (ii) distinguish abnormalities of *TP53* *versus* *ATM* and (iii) enable the identification of additional defects in the ATM-p53 pathway. Reversed transcriptase multiplex ligation-dependent probe amplification (RT-MLPA) was used to measure ATM and/or p53-dependent genes at the RNA level following DNA damage using irradiation. Here, we showed that this assay is able to identify and distinguish three subgroups of CLL tumors (i.e., *TP53*-defective, *ATM*-defective and WT) and is also able to detect additional samples with a defective DDR, without molecular aberrations in *TP53* and/or *ATM*. These findings make the ATM-p53 RT-MLPA functional assay a promising prognostic tool for predicting treatment responses in CLL.

Chronic lymphocytic leukemia (CLL), the most common leukemia in the Western world, is characterized by an extremely variable clinical course. Some patients have stable disease for many years, whereas others rapidly progress with immediate need for chemotherapy. Thus far, cytogenetic aberrations of the pivotal regulators of the DNA-damage response (DDR) pathway, *TP53* and *ATM*, have been shown to provide the most powerful predictive information on clinical outcome and responsiveness to chemotherapy.^1, 2, 3, 4, 5^

Approximately 8% and 18% of CLL patients requiring frontline therapy harbor defects of *TP53* or *ATM*, respectively.^3, 6^ These frequencies increase when the disease progresses following initial therapies. Although *TP53* and *ATM* aberrations both lead to p53 dysfunction, there are substantial differences both at the clinical and at the cellular level that distinguish *TP53*-defective from *ATM*-defective CLL. Clinically, *TP53*-defective CLL is more aggressive, whereas *ATM*-defective CLL has a more prolonged clinical course.^4^ At the cellular level, *TP53*-disruptive CLL exhibits a complete absence of DNA-damage-induced apoptosis *in vitro*, whereas *ATM*-disruptive CLL retains a capacity for apoptosis after *in vitro*-induced DNA damage, though at a reduced level.^7, 8^ In addition, microarray analyses revealed that *TP53*- and *ATM*-mutant CLL share a defect in activating proapoptotic responses after DNA damage but are distinguished by major differences in activating prosurvival responses.^9^

In CLL, the majority of *TP53* defects consists of biallelic *TP53* defects (70%), that is, a *TP53* deletion (17p deletion) in one allele in conjunction with a *TP53* mutation in the other allele.^5^ In marked contrast, less than 40% of *ATM* deletions (11q deletion) coincide with an *ATM* mutation.^2^ Whereas monoallelic lesions of *TP53* (i.e., mutations or deletions) commonly lead to p53 dysfunction and impaired responses to chemotherapy,^5, 10^ only biallelic defects of *ATM* (i.e., mutation and deletion) usually result in impaired p53 response.^2, 4, 11^

Currently, detection of deletions *via* fluorescent *in situ* hybridization (FISH) of *TP53* and *ATM* is part of standardized clinical work-up in CLL. Analyses of mutations in *TP53* and *ATM*, although of additional clinical value,^11, 12^ are currently not standardized and challenging, especially for *ATM,* owing to its extreme gene size with lack of well-characterized mutations.^11, 13^ Particularly, not all sequence variants in *ATM* lead to pathogenic changes.^13^

In addition to *TP53* and *ATM* defects, chemoresistance might be a consequence of epigenetic and posttranscriptional factors or deregulations of other components of the DDR, because more than 50% of chemo-refractory CLL patients do not exhibit *TP53* or *ATM* aberrations.^5^

Therefore, functional read-outs of the ATM/p53 axis with the aim to screen for (i) *TP53* and *ATM* mutations, (ii) discrimination between *TP53* and *ATM* defects, and (iii) additional defects in the DDR resulting from mechanisms other than *TP53/ATM* mutation/deletion, might add clinically relevant information on the actual DDR and chemosensitivity. This type of functional determination could add substantial information to FISH analysis. It is clinically important to distinguish *TP53* from *ATM* defects, because specific treatments that selectively sensitize *ATM*-deficient tumor cells to killing are emerging.^14^ Previously, we showed that a reverse transcriptase multiplex ligation-dependent probe amplification (RT-MLPA) procedure that quantifies the expression levels of the p53 targets, *CDKN1A*, *BBC3* and *Bax*, in CLL cells following irradiation is able to determine p53 functionality.^15^ With the aim of identifying and distinguishing abnormalities of *TP53* *versus* *ATM* and enabling the identification of additional defects in the DDR, we developed a new RT-MLPA-based functional assay.

## Results

### Prediction of ATM/p53 mutational status using RT-MLPA

The RT-MLPA assay was performed on all (*n*=30) samples from the training cohort and showed upregulation of cluster I genes following ionizing irradiation (IR) in WT samples and impaired upregulation in *TP53/ATM*-defective CLL samples following IR, confirming earlier results from Stankovic *et al.*^9^ Additionally, most cluster II-IV genes discriminated between *TP53-* and *ATM*-defective CLL samples in their regulation following IR. Results for each probe are shown in Supplementary Figure 2 in terms of fold induction (FI; expression upon IR in comparison with non-IR). No differences were observed in the expression of any of the included genes in the absence of IR between the different CLL mutational subgroups (data not shown). Because our aim was to develop a highly accurate RT-MLPA assay, in further analyses, we selected those gene probes that robustly discriminated the mutational subgroups (i.e., WT *versus TP53/ATM*-mutated samples for cluster I genes and *TP53*-defective *versus* *ATM*-defective CLL for cluster II-IV genes). Probes were selected by level of significance that resulted from the comparison between the two respective groups. In case of identical *P*-values, probes with the largest change in FI factors between the two respective groups were selected (Supplementary Table 4). This resulted in a set of 10 probes, containing the following genes: cluster I genes: *FAS*, *Bax*, *BBC3*, *CDKN1 A*, *PCNA*, *FDXR*; cluster II genes: *NME1*; cluster III genes: *MYC*, *PYCR1* and cluster IV genes: *ACSM3*.

**Figure 1:**
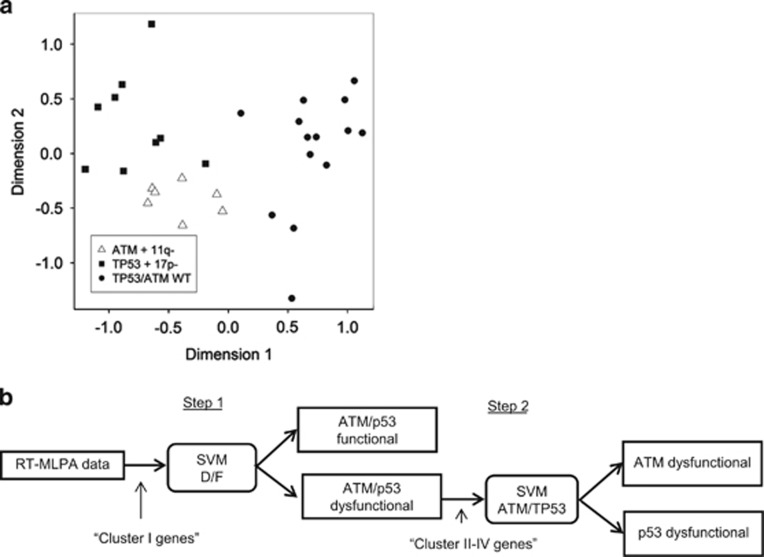
Design of statistical classifier based on the 10-gene panel. (**a**) Projection of the pairwise relationships among the samples included in the training cohort based on the FI of mRNA expression levels following irradiation and generated using multidimensional scaling analysis (MSA). Each patient is represented by a symbol, with the spatial proximity between any two symbols indicating the degree of similarity between the FI profiles of 10 selected genes (*FAS*, *Bax*, *BBC3*, *CDKN1A*, *FDXR*, *PCNA*, *NME1*, *MYC*, *PYCR1*, *ACSM3*) for the two corresponding patients. (**b**) Schematic overview of the two-stage construction of the support vector machine (SVM) classifiers, which allows classification of CLL samples into three different types of response, that is, ATM/p53-functional, p53-dysfunctional or ATM-dysfunctional based on the FIs of the cluster I-IV genes

To confirm that the 10-gene panel RT-MLPA could distinguish samples according to their ATM/p53 mutational status, we performed a multidimensional scaling analysis,^16^ a statistical method for exploring similarities or dissimilarities in data. Multidimensional scaling analysis showed clear separation between the WT, *TP53*-mutated and *ATM*-mutated cases, indicating that the 10-gene panel captured changes in gene expression associated with mutational status (Figure 1a). Next, based on the FI factors of the 10 selected genes, two support vector machine (SVM) classifiers were constructed to enable the classification of CLL samples into three different types of response, that is, ATM/p53 functional, p53-dysfunctional or ATM-dysfunctional. Models were constructed in a nested two-step approach. The first SVM predicts whether a sample is either ATM/p53 functional (F) or ATM/p53 dysfunctional (D) based on the FIs of the cluster I genes. The second SVM predicts whether an ATM/p53 dysfunctional sample is either ATM- or p53-dysfunctional based on the FIs of the cluster I-IV genes (Figure 1b). Internal cross-validation of the training set showed that the SVMs correctly classify ATM/p53-dysfunctional, p53-dysfunctional and ATM-dysfunctional patients. Of the WT patients, 13/14 (93%) were classified as functional and one as dysfunctional. All ATM-defective cases were correctly classified, whereas one out of nine TP53-defective samples was classified as ATM-dysfunctional. Note that these estimates are biased by the fact that for each gene in the panel, the most discriminative probe was selected based on the entire training cohort.

### Reproducibility of the RT-MLPA

To test for reproducibility of the RT-MLPA assay, we mixed RNA of CLL cells from all included *TP53/ATM* WT samples and analyzed this sample repeatedly in each experiment. In total, this sample was analyzed 23 times over a period of 3 years. The geometric mean with 95% confidence intervals for the FIs of individual genes are shown in Supplementary Table 5, illustrating that the RT-MLPA is highly robust with small 95% confidence intervals for all genes in the panel. Most importantly, all 23 replicate samples were classified consistently as ATM/p53 functional.

### Sensitivity of the RT-MLPA

In order to get an insight into the sensitivity of the functional assay in detecting subclones with *TP53* and *ATM* defects, we mixed varying proportions of RNA from CLL cells from patients with either biallelic *TP53* or biallelic *ATM* defects and a large clone size, with those from a patient with WT *TP53* and *ATM*. The assay was able to detect a functional defect when the defective *TP53* and *ATM* clone compromised around 35% and 45% of the sample, respectively (Supplementary Figure 3).

### Prediction of ATM/p53 mutational status in validation cohort; biallelic lesions

**Figure 2:**
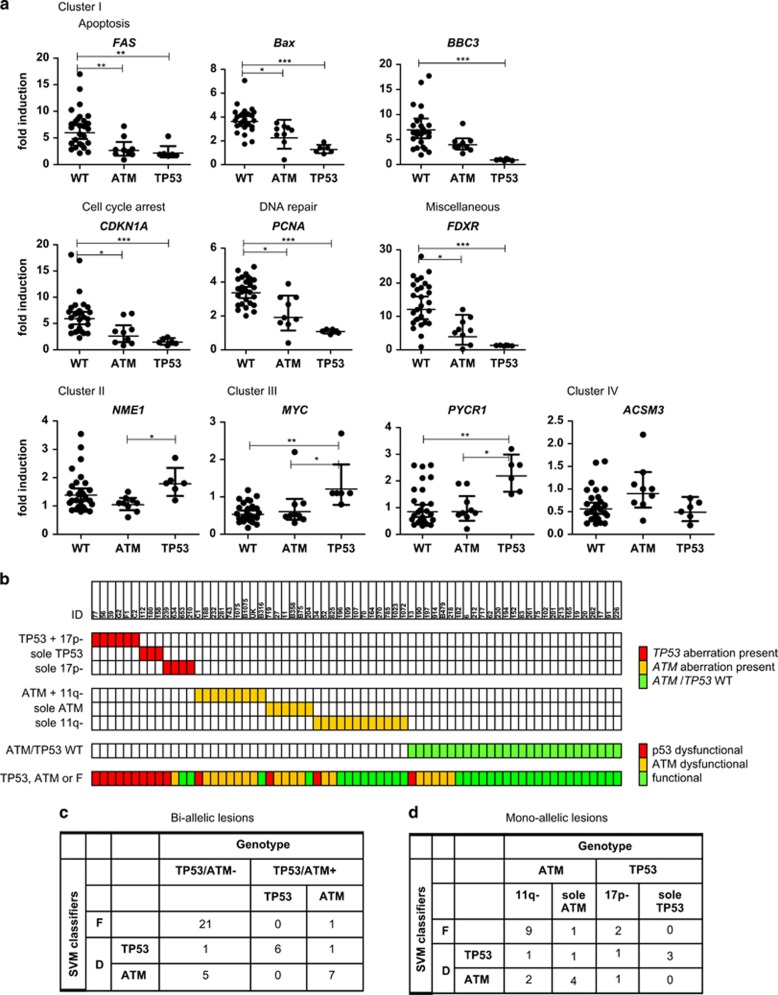
Validation of RT-MLPA assay and SVM classifiers in an independent validation cohort. (**a**) CLL cells of samples included in the validation set with clear genotypic characteristics, that is, *TP53/ATM* WT (*n*=27), biallelic *TP53* defects (*n*=6) or biallelic *ATM* defects (*n*=9) (i.e., mutation+deletion) were treated with or without irradiation (5 Gy) followed by measurement of mRNA expression levels using RT-MLPA. FI was calculated as the gene expression following irradiation divided by the gene expression level in the corresponding non-irradiated sample. Symbols represent individual patients. Geometric mean±95%CI within each group is shown. Significant differences in FI are presented as *0.01≤*P*<0.05; **0.001≤*P*<0.01; ***P<0.001 (Kruskal-Wallis test with Dunn's multiple comparison *post hoc* analysis). (**b**) Shown are the results of the SVM classifiers on the validation cohort (in lower line). Rows represent *TP53* and *ATM* aberrations, columns represent individual patients. In the upper three rows, color coding is based on: *TP53* aberrations (white, absence of *TP53* aberration; red, presence of *TP53* aberration). In the following three rows, color coding is based on: *ATM* aberrations (white, absence of *ATM* aberration; orange, presence of *ATM* aberration). In the following row, color coding is based on: absence of *TP53/ATM* aberrations (white, presence of *TP53* and/or *ATM* aberration; green, *TP53/ATM* WT). In the bottom row, color coding is based on results of SVM classifiers (red, p53-dysfunctional; orange, ATM-dysfunctional; green, p53/ATM-functional). (**c, d**) Contingency table for the classification of (**c**) bi-allelic lesions and (**d**) mono-allelic lesions

The classification models were validated on a separate cohort (validation cohort; Supplementary Table 1). First, CLL patients from the validation cohort with clear genotypic characteristics, that is, *TP53/ATM* WT (WT; *n*=27), biallelic *TP53* defects (*n*=6) or biallelic *ATM* defects (*n*=9) (i.e., mutation+deletion) were evaluated. Overall, patterns of response observed in the validation cohort were in agreement with those in the training cohort, with an intact upregulation of cluster I genes in WT and an impaired upregulation in *TP53*- and *ATM*-defective samples following irradiation. In addition, *TP53*-defective cases showed differential expression of cluster II-IV genes following IR in comparison with *ATM*-defective cases, with upregulation of *NME-1*, *MYC* and *PYCR1* (cluster II+III) and downregulation of *ACMS3*. (cluster IV; Figure 2a).

SVM predictions on those samples revealed that all (6/6) *TP53*-defective (17p-+*TP53* mutation) samples were classified as p53-dysfunctional. Eight out of nine *ATM*-defective (11q-+*ATM* mutation) cases were classified as dysfunctional (i.e., seven ATM-dysfunctional and one p53-dysfunctional) and one *ATM*-defective sample was classified as functional. Of the WT patients, 21/27 (78%) were classified as functional, whereas 6/27 (22%) and 1/27 (3.7%) were assigned as ATM-dysfunctional and p53-dysfunctional, respectively (Figures 2b and c). In summary, a high percentage of patients with *TP53/ATM* defects were classified as dysfunctional (sensitivity of 93%), with a high sensitivity for *TP53*-defects (100%) and relatively high sensitivity for *ATM*-defects (78% Figure 2c). In contrast, a relatively high percentage of WT patients were classified as dysfunctional, resulting in a specificity of 78%.

### Prediction of ATM/p53 mutational status in samples harboring monoallelic lesions

Next, monoallelic lesions were analyzed, that is, samples with sole *TP53* mutation (*n*=3), sole 17p deletion (*n*=4), sole *ATM* mutation (*n*=6) and sole 11q deletion (*n*=12) (Figures 2b and d). All (3/3) sole *TP53*-mutated samples were classified as p53-dysfunctional, whereas one out of four samples with a 17p deletion was classified as p53-dysfunctional (Figure 2d). The remaining three 17p-deleted samples were classified as ATM-dysfunctional (*n*=1) and functional (*n*=2), respectively. The two samples that were classified as functional harbored the deletion in only 15 and 50% of the cells, whereas the two samples which were classified as dysfunctional harbored the deletion in 80 and 96% of cells, suggesting a possible correlation between the degree of p53-(dys)functionality and clone size. With respect to monoallelic *ATM* aberrations, four out of six sole *ATM*-mutated cases were classified as ATM-dysfunctional, whereas one case was classified as p53-dysfunctional and one as functional. Nine out of 12 sole 11q-deleted samples were classified as functional, whereas 2 displayed an ATM-dysfunctional and one a p53-dysfunctional response. There was no correlation between the clone size of cells harboring an 11q deletion and the degree of ATM-(dys)functionality (data not shown).

### *In vitro* responses to DNA-damaging agents of samples classified according to the RT-MLPA

**Figure 3:**
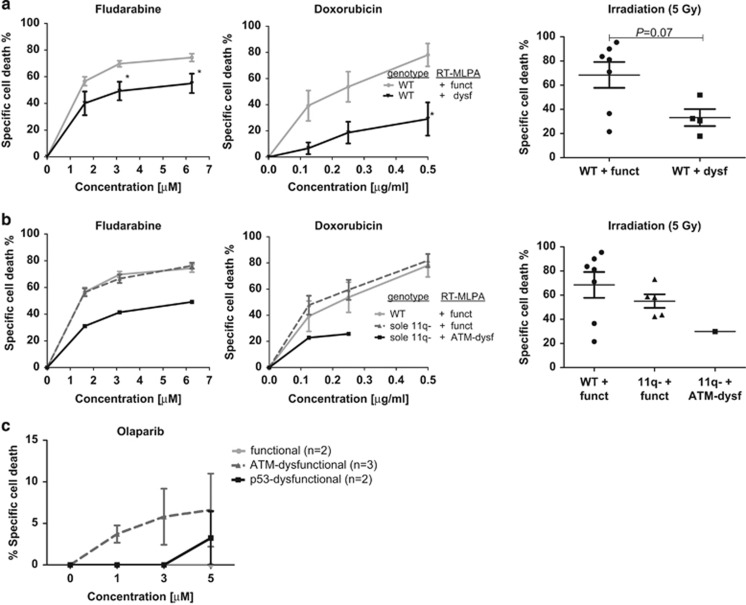
*TP53/ATM* WT CLL samples that were classified as ATM-dysfunctional display defective apoptotic responses to various DNA-damaging agents. (**a, b**) CLL cells of eight WT (nos. 6, 160, 647, 534, 854, 812, 912, 532) patients, which were classified as functional, and (**a**) four WT (nos. 13, 218, 190, 914) patients, which were classified as dysfunctional, and CLL cells of (**b**) five sole 11q deleted (nos. 785, 1023, 1072, 196, 270) patients, which were classified as p53/ATM-functional, and two sole 11q deleted (nos. 52, 825) patients, which were classified as ATM-dysfunctional, were treated with fludarabine or doxorubicin at increasing concentrations or irradiation (5 Gy). After 48 h, cell death was assessed by DIOC_6_/PI staining and specific cell death was calculated as described in the Materials and Methods section. Presented is mean±S.E.M. Significant differences in response compared with functional WT CLL cells at the same concentration are presented as *0.01≤*P*<0.05; **0.001≤*P*<0.01; ***P<0.001 (Mann-Whitney *U*-test). (**c**) CD40L/Il21 activated CLL cells of *n*=2 functional, *n*=3 ATM dysfunctional and *n*=2 p53 dysfunctional CLL samples classified according to the RT-MLPA were treated with olaparib at increasing concentrations. After 3 days, cell death was assessed by DIOC_6_/PI staining and specific cell death was calculated as described in the Materials and Methods section. Presented is mean±S.E.M.

In addition to *TP53* and *ATM* genetic defects, chemoresistance might be a consequence of other defects in the DDR, especially because more than 50% of chemo-refractory CLL patients do not exhibit *TP53* or *ATM* aberrations.^5^ Therefore, the RT-MLPA could be a very useful tool to detect ATM-p53 dysfunctional patients in the absence of *ATM* and/or *TP53* mutations. Interestingly, six WT samples were classified as dysfunctional (WT+dysf) according to the RT-MLPA-based SVM classifier. To determine whether these samples were indeed functionally affected, additional analyses of the *in vitro* apoptotic response to various DNA-damaging agents, that is, fludarabine, doxorubicin and irradiation were performed. In total, viable cells were available for *in vitro* testing of four WT+dysf samples. These were compared with WT samples (*n*=8) that were classified as functional (WT+funct). Interestingly, the WT+dysf samples showed significantly reduced apoptosis to all agents in comparison with the WT+funct samples (Figure 3a), indicating that defects in the DDR other than *TP53/ATM* aberrations are indeed present and probably responsible for the observed defective DNA-damage-induced apoptotic responses. In addition, apoptotic responses of the sole 11q-deleted samples, from which viable cells were available, were also examined. This revealed that the apoptotic responses of the samples that were classified as functional (*n*=5, 11q-+funct) according to the RT-MLPA-based SVM classifier were normal, whereas responses of the cases that were classified as ATM-dysfunctional (*n*=2, 11q-+ATM-dysf) were impaired (Figure 3b; note that for irradiation only one out of two 11q-+funct samples could be tested). These data indicate that the RT-MLPA assay is able to detect additional defects in the DDR resulting from mechanisms other than *TP53/ATM* aberrations and can potentially distinguish 11q-deleted samples in a dysfunctional and functional group.

It becomes increasingly important to distinguish *TP53* defects from *ATM* defects, because specific treatments that selectively sensitize *ATM*-deficient tumor cells to killing, such as the PARP-inhibitor olaparib, are emerging.^14^ To determine whether the RT-MLPA indeed can predict whether CLL cells respond to such specific treatments, cell death of samples classified as functional, ATM-dysfunctional or p53-dysfunctional was measured following olaparib treatment as described.^14^ We observed that olaparib only induced cell death in ATM-dysfunctional CLL cells following 3 *μ*M of olaparib (Figure 3c), which was in line with the observed levels of cell death as published by Weston *et al.*,^14^ showing that only *ATM* mutational CLL samples respond to olaparib.

## Discussion

Aberrations that involve the *TP53* or *ATM* gene affect the DDR pathway and are well-known adverse prognostic factors in CLL. Defects of the ATM-p53 pathway can also be caused by other mechanisms, such as polymorphisms in *MDM2*^17, 18^ and *CDKN1A,*^19^ hypermethylation of the *TP53* promotor^20^ or by novel recurrent mutations, such as those described recently for the *SAMHD1* gene.^21^ Identifying and distinguishing *TP53* and *ATM* defects has become increasingly relevant as specific treatments for *TP53-* and *ATM*-deficient tumors are emerging. However, mutational analyses of *TP53* and *ATM* in particular are challenging and not yet standardized. The aim of this study was to assess whether functional analysis of the ATM-p53 axis using a newly designed ATM-p53 functional assay could (i) detect *TP53* and/or *ATM* aberrations, (ii) distinguish *TP53* defects from *ATM* defects and (iii) enable the identification of additional defects in the ATM-p53 pathway.

In this study, we developed an RT-MLPA-assay that included genes differentially expressed upon irradiation between (i) WT and *TP53/ATM*-mutant CLL, and between (ii) *TP53-*mutant and *ATM*-mutant CLL. The RT-MLPA assay was subsequently evaluated in a training cohort with CLL samples with known *TP53* and *ATM* status, and support vector machine classifiers were constructed based on the FIs upon irradiation for a 10-gene panel. The RT-MLPA assay and SVM classifiers were validated in a separate validation cohort. CLL samples with clear genotypic characteristics (i.e., biallelic defects) were assigned with a high degree of confidence to one of the three categories with sensitivities of 93%, 100% and 78% for *TP53/ATM* WT, biallelic *TP53*-defective and biallelic *ATM*-defective samples, respectively. Thus, the RT-MLPA can both identify and distinguish biallelic *TP53* and *ATM* defects with a high degree of confidence.

Interestingly, a substantial number of WT samples were classified as ATM-dysfunctional (22%, 6/27), which might be cases that harbor other defects in the ATM-p53 pathway than aberrations of *TP53* and *ATM*. This is corroborated by the fact that apoptotic responses to various DNA-damaging agents were affected in the four cases that were further evaluated. Mutational analysis to uncover an underlying mechanism that could be involved in the observed defective DNA-damage-induced responses showed that two cases carried an *SF3B1* mutation,^22^ whereas the underlying defects in the other two samples remains elusive. These cases underscore the clinically highly relevant divergence between determination of ATM/p53 status by functional testing and by mutational analysis.

Samples with mono-allelic defects were not included in the training cohort and results were therefore more ambiguous. Mono-allelic deletions (i.e., sole 17p deletion and sole 11q deletion) were often classified as functional, whereas mono-allelic mutations (i.e., sole *TP53* mutation and sole *ATM* mutation) were often classified as p53-dysfunctional and ATM-dysfunctional, respectively. CLL samples with a sole 17p deletion are likely to be classified as functional by the RT-MLPA owing to a low clone size (± <40–50%). This is in agreement with other available p53-function assays.^10, 23, 24^ Sole 11q-deleted samples were often classified as functional (9/12), however, this was not clone-size dependent, as there was no correlation between the percentage of deleted cells and the degree of ATM-(dys)functionality. It is more likely that these samples were labeled functional owing to redundancy of the ATM-kinase activity in the remaining allele, in line with previous studies showing that CLL samples with both ATM alleles affected (either deletion and mutation or two mutations) lack ATM activity, while patients with monoallelic lesions may have preserved ATM function.^1, 2^ Although the number of investigated samples was low, the classification of the sole 11q-deleted samples according to the RT-MLPA indeed seems to be correct, because additional testing of apoptotic responses showed that two sole 11q-deleted samples that were classified as ATM-dysfunctional displayed impaired apoptotic responses, while the samples that were classified as functional showed intact apoptotic responses. Why 3 out of 12 sole 11q-deleted samples are classified as dysfunctional and the remaining ones as functional remains to be elucidated, but could be because of the involvement of currently unknown associated mutations or other factors.

Over the past years, several functional assays have been developed to test p53/ATM functionality, such as *MIR34a*, RT-PCR_*CDKN1A* and FACSp53-p21.^10, 18, 24, 25^ The majority of these functional assays were designed to assess p53 functionality and not to detect *ATM* defects specifically nor to distinguish *TP53* defects from *ATM* defects. In none of these studies, mutational analysis of *ATM* was performed. Some functional assays have been developed with the aim to identify and distinguish *TP53*- and *ATM*-defective tumors.^11, 26, 27^ One such assay is based on monitoring p53 and p21 accumulation after cell exposure to etoposide and nutlin-3a enabling the differentiation of *TP53* and *ATM* defects using flow cytometry.^26^ An alternative assay, based on measuring *CDKN1A* levels by RT-PCR following fludarabine and doxorubicin treatment, was primarily designed for *ATM* function testing and can also distinguish between *TP53* and *ATM* defects.^11^ As we have previously published, also cell death following DNA-damaging agents can distinguish a group of functional WT samples from a group of *ATM*-mutated and a group of *TP53*-mutated samples;^22^ however, this method seems less suitable to functionally test samples at the individual level to predict (dys)functionality, as exemplified in Figure 3a showing that two out of seven WT samples showed small percentages of cell death following irradiation, comparable with the percentage of cell death seen in dysfunctional patients. Finally, promising results were shown for the detection of *ATM* defects by measuring the percentage of mitotic cells with p53 localization at the centrosome.^27^ So far, these assays have not been validated in a separate cohort using the cutoff values determined in the initial study population, which is an important component of biomarker development.

A potential limitation of functional testing, not only in our study, but also in other studies evaluating p53 functional assays, is that small clones can be missed.^10, 23, 24^ This is especially important, because there is emerging evidence that the presence of mutations in subclones or in very small clones impact patient outcome, leading to reduced survival.^28, 29^ Another potential limitation is that functional testing usually needs viable cells with high purity. In case the viability of cells is low (<50%), the RT-MLPA functional assay is not reliable and WT samples could incorrectly be classified as dysfunctional.

In conclusion, the newly designed ATM-p53 RT-MLPA assay is able to distinguish three subgroups of CLL tumors (i.e., TP53-defective, ATM-defective and WT) and was also able to detect additional samples with a functional defective DDR, without molecular defects of *TP53* and/or *ATM*. This indicates that the ATM-p53 RT-MLPA might not only be of additional clinical value over FISH to screen for mutations of *TP53* and *ATM* instead of sequencing, but might also be useful for screening of other defects in the DDR pathway in addition to *ATM* and/or *TP53* aberrations. Whether the newly developed ATM-p53 RT-MLPA assay also predicts for clinical outcome in addition to the molecular status of *ATM* and/or *TP53* needs to be further evaluated in large clinical prospective studies.

## Materials and Methods

### Patient and samples

A cohort of 30 CLL patients from the Academic Medical Center, Amsterdam, the Netherlands and from the Central European Institute of Technology (CEITIC), Brno, Czech Republic, was enrolled in this study and utilized to set-up the RT-MLPA functional assay (training cohort). An independent second cohort consisted of 67 CLL patients included in the HOVON68 clinical trial,^30^ which was further enriched for patients with *TP53* and *ATM* aberrations from CEITEC (validation cohort). Clinical and genotypic characteristics are described in Supplementary Table 1. For further details on the training cohort, see also Supplementary Methods and Supplementary Figure 1. The study was conducted in accordance with the Declaration of Helsinki and written informed consent was obtained from all patients. Diagnosis of CLL was assessed according to IWCLL-NCI Working Group criteria. Peripheral blood mononuclear cells were isolated and frozen as described earlier.^31^ After thawing, CLL cells were enriched, in case CD19/CD5 purity was below 90%, *via* negative depletion using *α*-CD3 (CLB-T3/4,1,nr70,1x1), *α*-CD14 (CLB-mon/1,nr143,8G3) and *α*-CD16 (CLB-FCRgran1,nr142,5D2) (CLB, Amsterdam, the Netherlands) as described.^31^ Samples with a cell viability <50%, 16 h after thawing, determined by 3,3-dihexyloxacarbocyanine iodide (Invitrogen, Carlsbad, CA, USA) and propidium iodide (Sigma-Aldrich, St. Louis, MO, USA) using flow cytometry as described,^31^ were excluded from the analysis.

### Molecular analyses of *TP53* and *ATM*

Deletions at the 11q22-q23 (*ATM*), 17p13 (*TP53*), 13q14 loci and trisomy of chromosome 12 were detected by FISH by using locus-specific probes (Abott Vysis Inc., Des Plaines, IL, USA or MetaSystems, Altlussheim, Germany). *TP53* (ex4-10) mutational analysis was performed by next generation sequencing using the GS Junior 454 platform (Roche, Basel, Switzerland)^32^ or by Sanger sequencing using standard conditions as described previously.^5^ *ATM* (ex4-65) was analyzed by either Sanger sequencing and ATM functional analysis assessing irradiation induced phosphorylation of ATM targets or by resequencing microarray and direct sequencing as described previously.^1, 11^

### Apoptosis induction by various DNA-damaging agents

Thawed CLL cells were cultured at a concentration of 1.5 × 10^6^/ml in the presence of fludarabine or doxorubicin (Sigma-Aldrich) or following exposure to irradiation (5 Gy), for 48 h at 37 °C. For olaparib studies, thawed CLL cells were stimulated with a CD40/IL-21 culture system for 4 days. Briefly, murine fibroblast cells (3T3) expressing CD40L were irradiated (30 Gy) and divided over 48-well plates. After attachment of the fibroblasts, 0.5 × 10^6^ CLL cells were seeded into each well with 25 ng/ml IL-21 (Gibco, Carlsbad, CA, USA; Invitrogen) in a total volume of 500 *μ*l per well and incubated at 37 °C for 4 days. Cells were then harvested and replated on CD40L-expressing 3T3 cells and incubated with olaparib (AstraZeneca, London, UK) at various doses in the presence of IL-21 for an additional 3 days. Apoptosis was measured by flow cytometry as previously described.^31^ Specific cell death was calculated as (%apoptosis_treated cells_−% apoptosis_untreated cells_)/%viable_untreated cells._

### p53 and ATM target gene induction

CLL cells were treated with or without irradiation (5 Gy) and cultured for 16 h at a concentration of 5.0 × 10^6^ cells/ml at 37 °C. RNA was isolated using an RNA-isolation kit (Sigma-Aldrich) according to the manufacturer's instructions and subsequently RT-MLPA was performed.

### Design of p53/ATM RT-MLPA assay

A new RT-MLPA probe set (R016-X2, MRC-Holland), which included several p53 and ATM target genes was designed. Genes were selected based on the results of an earlier microarray study.^9^ In that study, genes differentially expressed between WT (*n*=5), *TP53*-mutated (*n*=5) and *ATM*-mutated (*n*=6) CLL samples in response to DNA-damage using IR were determined and classified into four major clusters. Cluster I represented genes normally upregulated in response to IR in the presence of functionally active ATM and p53, whereas clusters II-IV represented genes whose transcription was upregulated after IR in the presence of functionally inactive p53 (cluster III) or whose transcription was not upregulated or not downregulated in the presence of inactive ATM (cluster II and IV, respectively; Supplementary Table 2). The previous RT-MLPA kit^15^ contained three cluster I genes and no genes from the other clusters. In the current RT MLPA assay, additional cluster I genes and at least two genes for each of the clusters II-IV were added. See Supplementary Methods for further details on the selection of genes and design of the RT-MLPA kit. For each gene, at least two hemiprobes were designed, which, if possible, span exon boundaries, precluding the detection of potentially contaminating genomic DNA. Furthermore, four housekeeping genes, that is, *Diablo*, *Aif*, *Gusb* and *Parn*, were included. The housekeeping genes were selected from an earlier RT-MLPA assay design (apoptosis kit R011-C1, MRC-Holland), because their expression was not influenced by irradiation as established by geNorm software.^33^ Target genes and probes are listed in Supplementary Table 3.

### Reversed transcriptase multiplex ligation-dependent probe amplification

For preparation of the M13-derived MLPA probe oligonucleotides, reaction conditions and detailed further information on RT-MLPA in general (see Eldering *et al.*^34^ and MRC Holland website). Expression levels in a sample were normalized to the geometric mean of the expression of the four housekeeping genes in the same sample. For each patient, FIs were calculated by dividing the expression level in the irradiated sample by the expression level in the corresponding non-irradiated sample.

### Statistical analysis

A non-parametric Mann-Whitney U test was used for comparison of two independent groups in the training cohort and a non-parametric Kruskal-Wallis test with Dunn's multiple comparison *post hoc* analysis was used for comparison of multiple groups in the validation cohort. Correlations were analyzed by Spearman's rank correlation test. A *P*-value <0.05 was considered statistically significant.

## Abbreviations

CLL: chronic lymphocytic leukemia
TP53: tumor protein p53
ATM: ataxia telangiectasia mutated
RT-MLPA: reversed transcriptase-multiplex ligation-dependent probe amplification
WT: wild type
DNA: deoxyribonucleic acid
DDR: DNA-damage response
FISH: fluorescence *in situ* hybridization
CDKN1A: cyclin-dependent kinase N1A
BBC3: bcl-2 homology domain 3
HOVON: Stichting Hemato-Oncologie voor Volwassenen Nederland
iwCLL-NCI: International Workshop on Chronic Lymphocytic Leukemia - National Cancer Institute
DIOC6: 3,3-dihexyloxacarbocyanine iodide
PI: propidium iodide
RNA: ribonucleic acid
IR: ionizing irradiation
PCNA: proliferating cell nuclear antigen
FDXR: ferredoxin reductase
NME1: nm23-H1 gene
PYCR1: pyrroline-5-carboxylate reductase 1
ACSM3: acyl-CoA synthetase medium-chain family member 3
MDM2: mouse double minute 2 homolog

## References

[bib1] Austen B Powell JE Alvi A Edwards I Hooper L Starczynski J Mutations in the ATM gene lead to impaired overall and treatment-free survival that is independent of IGVH mutation status in patients with B-CLL Blood 2005 106 3175 3182 16014569 10.1182/blood-2004-11-4516

[bib2] Austen B Skowronska A Baker C Powell JE Gardiner A Oscier D Mutation status of the residual ATM allele is an important determinant of the cellular response to chemotherapy and survival in patients with chronic lymphocytic leukemia containing an 11q deletion J Clin Oncol 2007 25 5448 5457 17968022 10.1200/JCO.2007.11.2649

[bib3] Dohner H Fischer K Bentz M Hansen K Benner A Cabot G p53 gene deletion predicts for poor survival and non-response to therapy with purine analogs in chronic B-cell leukemias Blood 1995 85 1580 1589 7888675

[bib4] Skowronska A Parker A Ahmed G Oldreive C Davis Z Richards S Biallelic ATM inactivation significantly reduces survival in patients treated on the United Kingdom Leukemia Research Fund Chronic Lymphocytic Leukemia 4 trial J Clin Oncol 2012 30 4524 4532 23091097 10.1200/JCO.2011.41.0852

[bib5] Zenz T Eichhorst B Busch R Denzel T Häbe S Winkler D TP53 mutation and survival in chronic lymphocytic leukemia J Clin Oncol 2010 28 4473 4479 20697090 10.1200/JCO.2009.27.8762

[bib6] Stankovic T Weber P Stewart G Bedenham T Murray J Byrd PJ Inactivation of ataxia telangiectasia mutated gene in B-cell chronic lymphocytic leukaemia Lancet 1999 353 26 29 10023947 10.1016/S0140-6736(98)10117-4

[bib7] Pettitt AR Sherrington PD Stewart G Cawley JC Taylor AM Stankovic T p53 dysfunction in B-cell chronic lymphocytic leukemia: inactivation of ATM as an alternative to TP53 mutation Blood 2001 98 814 822 11468183 10.1182/blood.v98.3.814

[bib8] Stankovic T Stewart GS Fegan C Biggs P Last J Byrd PJ Ataxia telangiectasia mutated-deficient B-cell chronic lymphocytic leukemia occurs in pregerminal center cells and results in defective damage response and unrepaired chromosome damage Blood 2002 99 300 309 11756185 10.1182/blood.v99.1.300

[bib9] Stankovic T Hubank M Cronin D Stewart GS Fletcher D Bignell CR Microarray analysis reveals that TP53- and ATM-mutant B-CLLs share a defect in activating proapoptotic responses after DNA damage but are distinghuished by major differences in activating prosurvival responses Blood 2004 103 291 300 12958068 10.1182/blood-2003-04-1161

[bib10] Mohr J Helfrich H Fuge M Eldering E Bühler A Winkler D DNA damage-induced transcriptional program in CLL: biological and diagnostic implications for functional p53 testing Blood 2011 117 1622 1632 21115975 10.1182/blood-2010-08-300160

[bib11] Navrkalova V Sebejova L Zemanova J Kminkova J Kubesova B Malcikova J ATM mutations uniformly lead to ATM dysfunction in chronic lymphocytic leukemia: application of functional test using doxorubicin Haematologica 2013 98 1124 1131 23585524 10.3324/haematol.2012.081620 PMC3696617

[bib12] Pospisilova S Gonzalez D Malcikova J Trbusek M Rossi D Kater AP ERIC recommendations on TP53 mutation analysis in chronic lymphocytic leukemia Leukemia 2012 26 1458 1461 22297721 10.1038/leu.2012.25

[bib13] Stankovic T Skowronska A The role of ATM mutations and 11q deletions in disease progression in chronic lymphocytic leukemia Leuk Lymphoma 2014 55 1227 1239 23906020 10.3109/10428194.2013.829919

[bib14] Weston VJ Oldreive CE Skowronska A Oscier DG Pratt G Dyer MJ The PARP inhibitor olaparib induces significant killing of ATM-deficient lymphoid tumor cells *in vitro* and *in vivo* Blood 2010 116 4578 4587 20739657 10.1182/blood-2010-01-265769

[bib15] Mous R Jaspers A Luijks DM Mellink CH van Oers MH Kater AP Detection of p53 dysfunction in chronic lymphocytic leukaemia cells through multiplex quantification of p53 target gene induction Leukemia 2009 23 1352 1355 19340003 10.1038/leu.2009.71

[bib16] Cox TF Cox MAAMultidimensional Scaling. Boca Raton: Chapman & Hall2nd edition. 2011

[bib17] Zent CS Time to test CLL p53 function Blood 2010 115 4154 4155 20508168 10.1182/blood-2010-02-268482

[bib18] Asslaber D Pinon JD Seyfried I Desch P Stöcher M Tinhofer I microRNA-34a expression correlates with MDM2 SNP309 polymorphism and treatment-free survival in chronic lymphocytic leukemia Blood 2010 115 4191 4197 20089965 10.1182/blood-2009-07-234823

[bib19] Johnson GG Sherrington PD Carter A Lin K Liloglou T Field JK A novel type of p53 pathway dysfunction in chronic lymphocytic leukemia resulting from two interacting single nucleotide polymorphisms within the p21 gene Cancer Res 2009 69 5210 5217 19491257 10.1158/0008-5472.CAN-09-0627

[bib20] Valganon M Giraldo P Agirre X Larráyoz MJ Rubio-Martinez A Rubio-Felix D p53 Aberrations do not predict individual response to fludarabine in patients with B-cell chronic lymphocytic leukaemia in advanced stages Rai III/IV Br J Haematol 2005 129 53 59 15801955 10.1111/j.1365-2141.2005.05405.x

[bib21] Clifford R Louis T Robbe P Ackroyd S Burns A Timbs AT SAMHD1 is mutated recurrently in chronic lymphocytic leukemia and is involved in response to DNA damage Blood 2014 123 1021 1031 24335234 10.1182/blood-2013-04-490847 PMC3924925

[bib22] te Raa GD Derks IA Luijks DM van Laar J Monsuur H Oldreive C SF3B1 mutations in CLL are equivalent to p53/ATM dysfunction and cause defective puma upregulation in response to chemotherapy Blood (ASH Annual Meeting Abstracts) 8–11 December 2012Abstract 711.

[bib23] Carter A Lin K Sherrington PD Atherton M Pearson K Douglas A Imperfect correlation between p53 dysfunction and deletion of TP53 and ATM in chronic lymphocytic leukaemia Leukemia 2006 20 737 740 16437137 10.1038/sj.leu.2404120

[bib24] Lin K Adamson J Johnson GG Carter A Oates M Wade R Functional analysis of the ATM-p53-p21 pathway in the LRF CLL4 trial: blockade at the level of p21 is associated with short response duration Clin Cancer Res 2012 18 4191 4200 22675167 10.1158/1078-0432.CCR-11-2936

[bib25] Mraz M Malinova K Kotaskova J Pavlova S Tichy B Malcikova J miR-34a, miR-29c and miR-17-5p are downregulated in CLL patients with TP53 abnormalities Leukemia 2009 23 1159 1163 19158830 10.1038/leu.2008.377

[bib26] Best OG Gardiner AC Majid A Walewska R Austen B Skowronska A A novel functional assay using etoposide plus nutlin-3a detects and distinguishes between ATM and TP53 mutations in CLL Leukemia 2008 22 1456 1459 18200038 10.1038/sj.leu.2405092

[bib27] Prodosmo A De AA Nistico C Gabriele M Di Rocco G Monteonofrio L p53 centrosomal localization diagnoses ataxia-telangiectasia homozygotes and heterozygotes J Clin Invest 2013 123 1335 1342 23454770 10.1172/JCI67289 PMC3582149

[bib28] Landau DA Carter SL Stojanov P McKenna A Stevenson K Lawrence MS Evolution and impact of subclonal mutations in chronic lymphocytic leukemia Cell 2013 152 714 726 23415222 10.1016/j.cell.2013.01.019 PMC3575604

[bib29] Rossi D Khiabanian H Spina V Ciardullo C Bruscaggin A Famà R Clinical impact of small TP53 mutated subclones in chronic lymphocytic leukemia Blood 2014 123 2139 2147 24501221 10.1182/blood-2013-11-539726 PMC4017291

[bib30] Geisler CH van T' Veer MB Jurlander J Walewski J Tjønnfjord G Itälä Remes M Frontline low-dose alemtuzumab with fludarabine and cyclophosphamide prolongs progression-free survival in high-risk CLL Blood 2014 123 3255 3262 24735962 10.1182/blood-2014-01-547737

[bib31] Mackus WJ Kater AP Grummels A Evers LM Hooijbrink B Kramer MH Chronic lymphocytic leukemia cells display p53-dependent drug-induced Puma upregulation Leukemia 2005 19 427 434 15674362 10.1038/sj.leu.2403623

[bib32] Jethwa A Hullein J Stolz T Blume C Sellner L Jauch A Targeted resequencing for analysis of clonal composition of recurrent gene mutations in chronic lymphocytic leukaemia Br J Haematol 2013 163 496 500 24032483 10.1111/bjh.12539

[bib33] Vandesompele J De PK Pattyn F Poppe B Van Roy N De Paepe A Accurate normalization of real-time quantitative RT-PCR data by geometric averaging of multiple internal control genes Genome Biol 2002 3 RESEARCH0034 12184808 10.1186/gb-2002-3-7-research0034 PMC126239

[bib34] Eldering E Spek CA Aberson HL Grummels A Derks IA de Vos AF Expression profiling via novel multiplex assay allows rapid assessment of gene regulation in defined signalling pathways Nucleic Acids Res 2003 31 e153 14627843 10.1093/nar/gng153 PMC290288

