# Supplementary material for: Assessment of p53 and ATM functionality in chronic lymphocytic leukemia by multiplex ligation-dependent probe amplification
Source: Cell Death Dis. 2015 Aug 6;6(8):e1852–. doi: 10.1038/cddis.2015.223 (PMC4558513; doi:10.1038/cddis.2015.223)
Supplement: Supplementary Information [file cddis2015223x1.doc]

**Supplemental Methods**

**Composition of training cohort**

A training cohort of 30 CLL samples with known *TP53/ATM* molecular and functional characteristics was used to test the newly designed RT-MLPA. *TP53* and *ATM* molecular status was determined using FISH and sequencing. Only samples with biallelic *TP53* (*TP53* mutation + 17p deletion) and biallelic *ATM* (*ATM* mutation + 11q deletion) defects were included. In addition, *TP53*-mutant CLL samples were to a large extent derived from patients with chemorefractory disease. In total, the training cohort consisted of 14 *TP53*/*ATM* wild type (WT), 9 *TP53-* and 7 *ATM*-defective samples (Supplemental Table 1). Chemo-sensitivity *in vitro* was assessed by measuring induction of cell death at 48 hours of fludarabine treatment. Our results indicated three types of fludarabine responses (Supplemental Figure 1). This is in accordance with previous studies showing that exposure to irradiation led to intact apoptotic responses in WT (specific cell death of >70% following 6.25 µM fludarabine), a virtual absence of apoptosis in *TP53*-mutant CLL (specific cell death of <20% following 6.25 µM fludarabine) and a level of apoptosis that was intermediate between that of WT and *TP53*-mutant tumors, in *ATM*-mutant tumors^1;2^.

**Design of RT-MLPA kit: selection of genes and probes**

A new RT-MLPA probe set (R016-X2, MRC-Holland), which included several p53 and ATM target genes, was designed based on the results of an earlier microarray study^3^. We selected genes from the earlier published microarray study^3^ using the following criteria: (i) level of expression upon irradiation in comparison to non-irradiation (fold induction factor) of all samples within each group that were compared had to differ (i.e. WT vs. *TP53/ATM*-mutated for cluster I genes and *ATM* vs. *TP53* mutated samples for cluster II-IV genes) and (ii) sufficiently high expression in the non-irradiated WT samples. The latter was defined by an expression level of at minimum 20% lower than the expression level of *FAS* in the microarray, since we know from earlier experience with the RT-MLPA that the expression of *FAS* is just above the detection limit.

**Design of statistical classifiers**

Analyses were carred out in the statistical software package R (version 3.0.0) using package e1071 (version 1.6-1) and Bioconductor packages limma (version 3.16.8) and MCRestimate (version 2.16.0) ^4^. At most 1.5% of gene expression values were missing in any of the cohorts. Missing values were imputed by the minimal expression value for that gene in the other samples of the same condition from the same cohort. Subsequently, gene inductions were calculated by dividing the expression level in the irradiated sample by the expression in the corresponding non-irradiated sample and then log2-transformed. Multidimensional scaling analysis was performed on the genewise standardized log2-transformed fold induction values using the root-mean-square deviation (Euclidean) distance measure (function plotMDS, limma).

Two linear support vector machine (SVM) classifiers were constructed to enable the classification of CLL samples into three different types of response, i.e. ATM/p53 functional, p53-dysfunctional, or ATM-dysfunctional. Models were constructed in a nested two-step approach. The first SVM predicts whether a sample is either ATM/p53 functional or ATM/p53 dysfunctional based on the log2-transformed fold inductions of the cluster I genes. The second SVM predicts whether an ATM/p53 dysfunctional sample is either ATM- or p53-dysfunctional based on the log2-transformed fold inductions of the cluster I-IV genes (Figure 1B, main text). Predictive performance on the training cohort was estimated using nested cross-validation (5-fold inner cross-validation, 3-fold outer cross-validation, with ten repeats). Optimal values for the ‘cost’ parameter were selected in the inner cross-validation loop and predictive performance was calculated in the outer cross-validation loop (function MCRestimate). In order to allow for probability predictions, the two final models were fit on the training cohort using the function ‘tune’ (package e1071) with 5-fold inner cross-validation to select optimal values for the ‘cost’ parameter. Resulting SVMs were applied to a validation cohort that was not used in the construction of the classification models.

Supplemental

**Supplemental Legends**

**Supplemental Figure 1. Apoptotic responses upon fludarabine of CLL samples included in the training cohort.** CLL cells of *TP53*/*ATM* WT, *ATM*-mutant and *TP53*-mutant CLL samples included in the training cohort were treated with fludarabine 6.25 µM for 48 hours and cell death was assessed by DIOC_6_/PI staining using flowcytometry. Specific cell death was calculated as described in the Materials and Methods section. Horizontal bars, boxes, whiskers and dots represent median, 25%/75% quartiles, range and outliers, respectively. Significant differences are presented as *0.01≤*P*<0.05; **0.001≤*P*<0.01; ****P*<0.001 (Mann-Whitney U test).

**Supplemental Figure 2. Selection of genes and probes included in the RT-MLPA assay.** CLL cells of samples included in the training cohort: 14 *TP53*/*ATM* WT, 9 *TP53* and 7 *ATM* mutated patients were treated with or without irradiation (5Gy) followed by measurement of mRNA expression levels using RT-MLPA. Fold induction was calculated as the gene expression level following irradiation divided by the gene expression level in the corresponding non-irradiated sample. Symbols represent individual patients. Geometric mean±95%CI within each group is shown. Significant differences in fold induction are presented as *0.01≤*P*<0.05; **0.001≤*P*<0.01; ****P*<0.001 (Mann-Whitney U test).

**Supplemental Figure 3. RT-MLPA response in heterogeneous *TP53*/WT and *ATM*/WT samples.**

RNA obtained from one *TP53*/*ATM* WT sample, one *TP53-*mutated and one *ATM*-mutated sample was mixed in different ratios as indicated. SVM-based classification of the mRNA expression levels measured using RT-MLPA was performed for each clone size. Shown is the probability of *TP53*/*ATM*-dysfunctionality as determined by the SVM classifier versus the percentage of defective *TP53* or *ATM* mRNA present. Hatched lines indicate the cut-off between functional and dysfunctional. One outlier is shown in the figure at 70% *TP53* MUT which was considered to be a pipetting mistake.
