# Supplementary material for: Assessment of p53 and ATM functionality in chronic lymphocytic leukemia by multiplex ligation-dependent probe amplification
Source: Cell Death Dis. 2015 Aug 6;6(8):e1852–. doi: 10.1038/cddis.2015.223 (PMC4558513; doi:10.1038/cddis.2015.223)
Supplement: Supplementary Figures [file cddis2015223x2.ppt]

## Slide 1
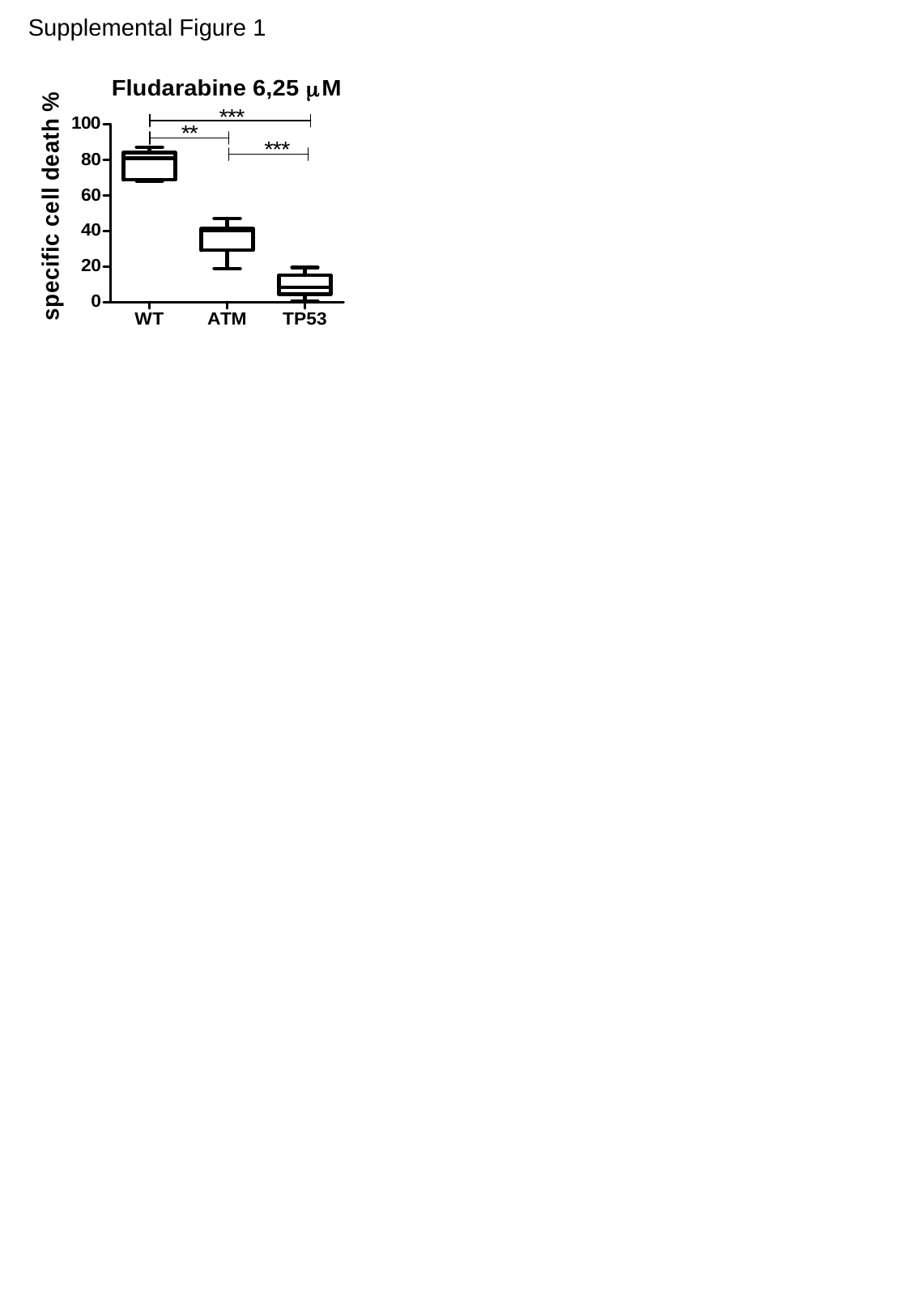

Supplemental Figure 1

## Slide 2
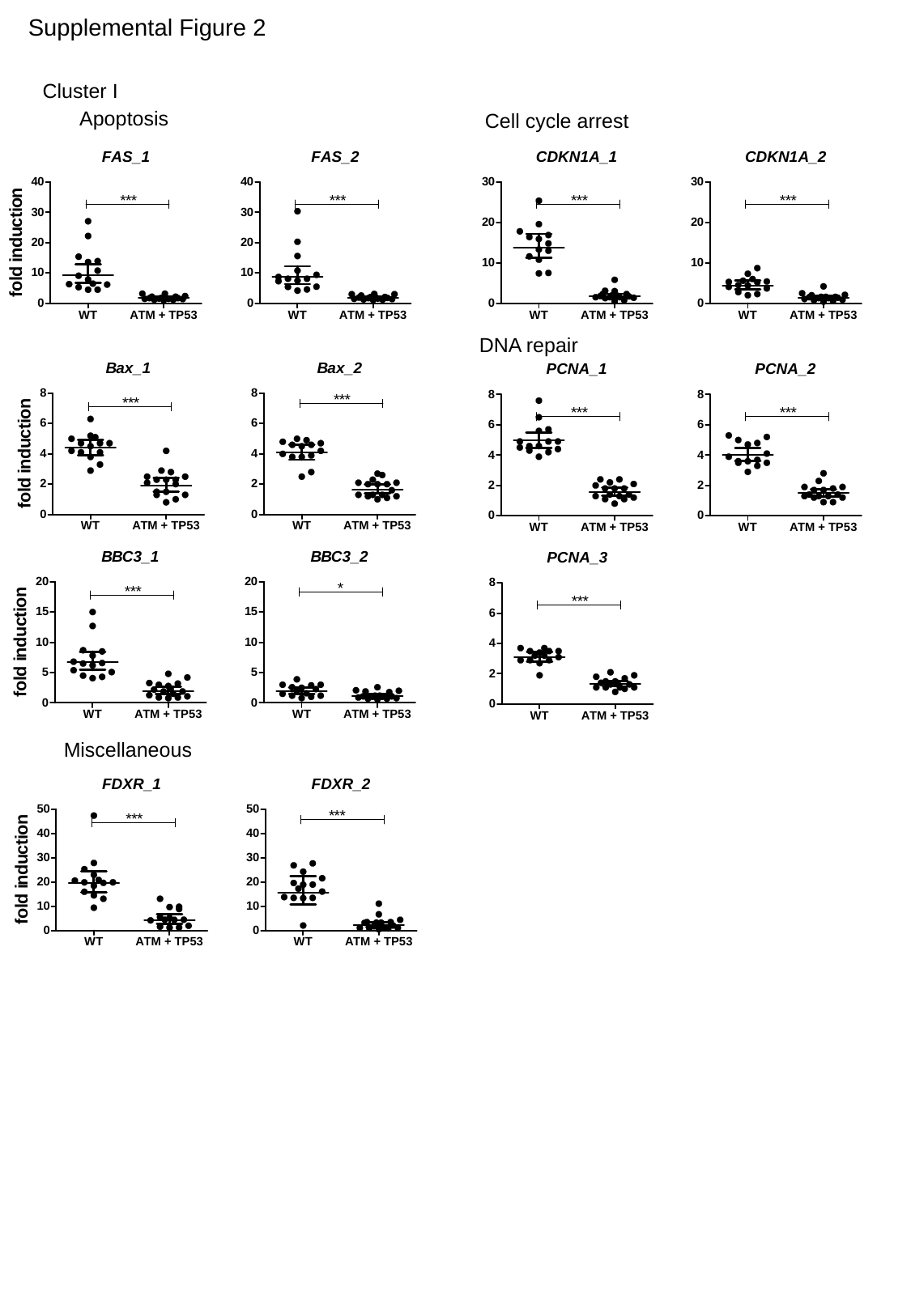

Supplemental Figure 2
Cluster I
Apoptosis
Cell cycle arrest
DNA repair
Miscellaneous

## Slide 3
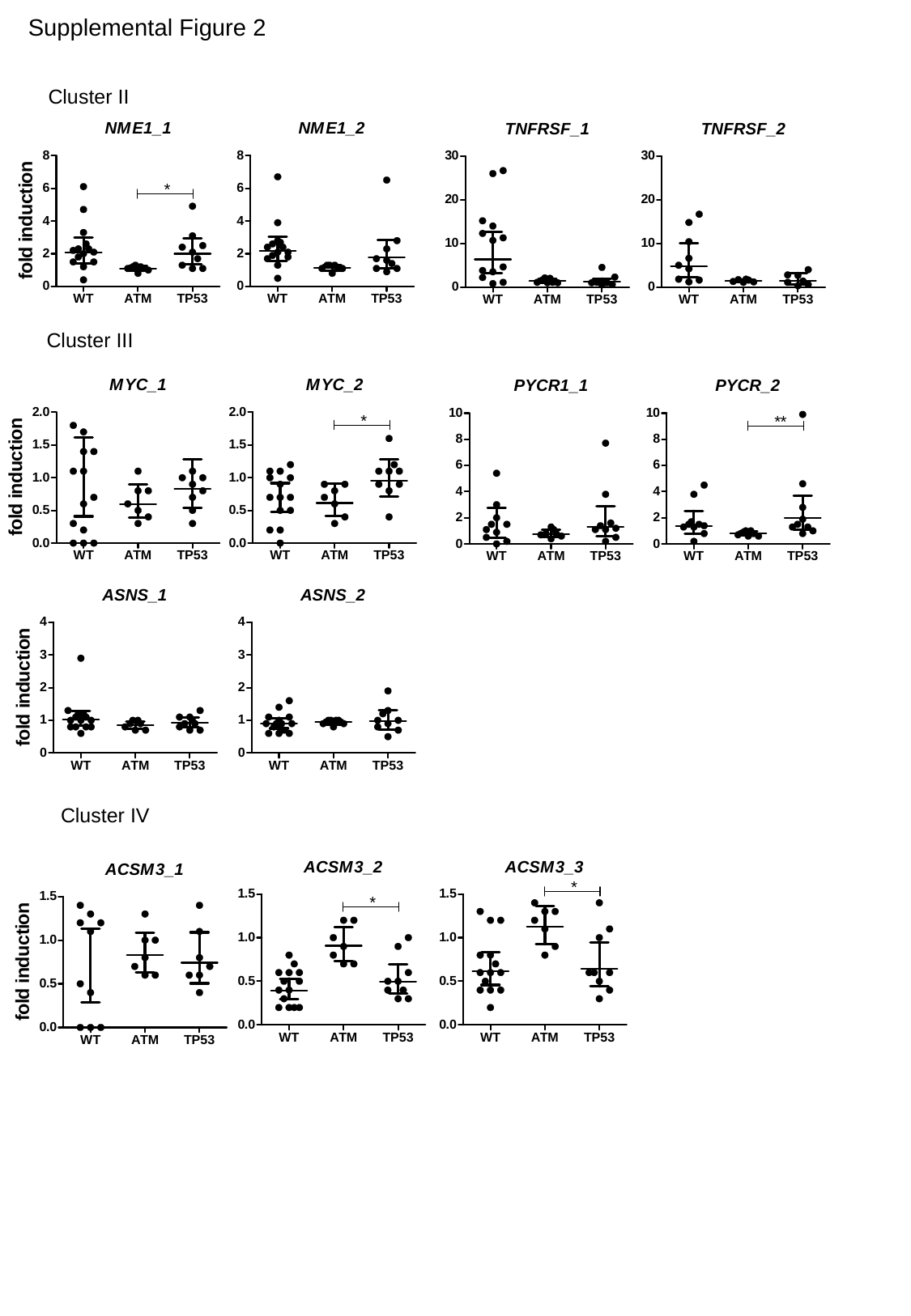

Supplemental Figure 2
Cluster II
Cluster III
Cluster IV

## Slide 4
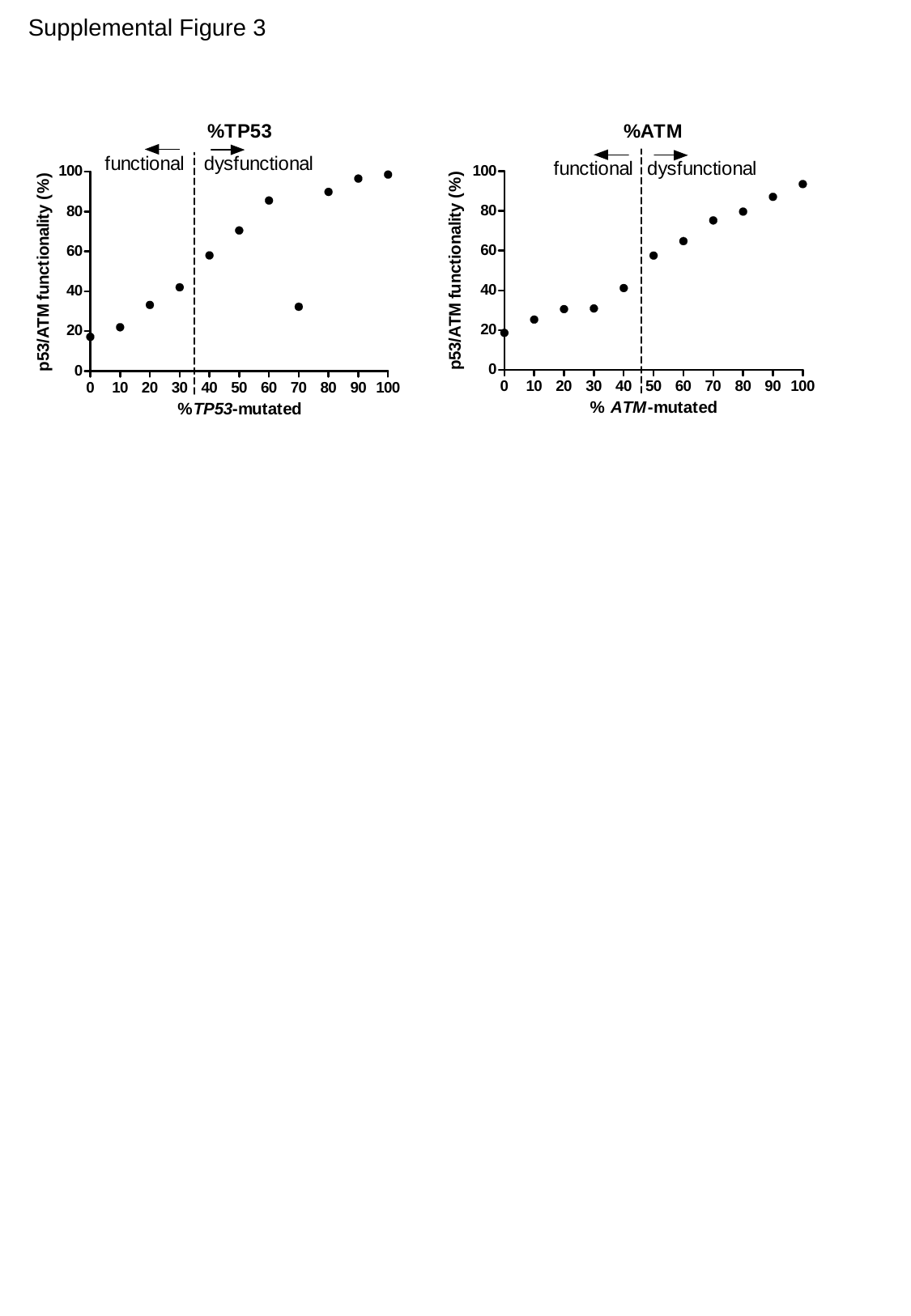

Supplemental Figure 3
