# Supplementary material for: Assessment of p53 and ATM functionality in chronic lymphocytic leukemia by multiplex ligation-dependent probe amplification
Source: Cell Death Dis. 2015 Aug 6;6(8):e1852–. doi: 10.1038/cddis.2015.223 (PMC4558513; doi:10.1038/cddis.2015.223)
Supplement: Supplementary Tables [file cddis2015223x3.doc]

**Supplemental Table 1. Patient characteristics and type of mutations from patients included in training cohort and validation cohort.**

**Training cohort**

| No. | Age | Rai-stage | IGHV mutation | 17p, 11q (%)* | Mutation  TP53 or ATM (%)** | Amino acid change | Nucleotide  change | Earlier treatment | Chemo refract in vivo |
| --- | --- | --- | --- | --- | --- | --- | --- | --- | --- |
| 605 | 36 | III | UM | 17p (87) | TP53 | Q317STOP | 949C>T | Chl, CVP, FCR, Al, R-CHOP | Yes (F) |
| 452 | 82 | IV | M | 17p (53) | TP53 | R273C | 817C>T | Chl | Yes (Chl) |
| 172 | 64 | I | UM | 17p (61) | TP53 | 730G>A | G244S | Chl | Yes (F) |
| 406 | 59 | I | UM | 17p (67) | TP53 | C135R | 403T>C | no | NA |
| 1028 | 58 | I | M | 17p (94) | TP53 | H178P | 533A>C | Chl, FCR, RDHAP | Yes (F) |
| 456 | 67 | II | M | 17p (94) | TP53 | C141Y | 422G>A | no | Yes (F)*** |
| 83_A | 33 | II | UM | 17p (83) | TP53 | R175H | 524G>A | Chl, FCR | Yes (F) |
| 649 | 69 | I | NA | 17p (77) | TP53 | D281G | 842A>G | no | Yes (F)*** |
| 701 | 73 |  | NA | 17p (45) | TP53 | C176Y | 527G>A | no | NA |
| 508 | 54 | IV | M | 11q (75) | ATM | E343fs | 1027delGAAA | no | NA |
| 316 | 46 | II | M | 11q (72) | ATM | P338L  S2494L | 1148C>T  7181C>T | Chl | No (Chl) |
| 13_A | 57 | I | UM | 11q (NA) | ATM | C2624fs  L2953Q  L55STOP | 7868insT  8858T>A  164T>A | no | NA |
| B296 | 70 | IV | UM | 11q (94) | ATM | L2427P | 7280T>C | FC, F | NA |
| B1043 | 77 | IV | UM | 11q (90) | ATM | C2488Y | 7463G>A | no | NA |
| B182 | 44 | III | UM | 11q (99) | ATM | Q1084STOP | 3250C>T | no | NA |
| B192 | 64 | IV | UM | 11q(UPD) | ATM | skipp. ex 10 | 902-1G>T | FC, Al | NA |
| 160 | 50 | I | UM | no | no |  |  | no |  |
| 854 | 79 | 0 | NA | no | no |  |  | no |  |
| 812 | 76 | I | M | no | no |  |  | Chl |  |
| 912 | 76 | 0 | UM | no | no |  |  | no |  |
| 534 | 42 | 0 | M | no | no |  |  | no |  |
| 647 | 58 | III | M | no | no |  |  | no |  |
| 532 | 78 | 0 | M | no | no |  |  | no |  |
| 156 | 68 | II | M | no | no |  |  | no |  |
| 166 | 50 | I | UM | no | no |  |  | no |  |
| 67 | 48 | I | M | no | no |  |  | no |  |
| 154 | 65 | III | M | no | no |  |  | no |  |
| 162 | 62 | III | M | no | no |  |  | no |  |
| 636 | 63 | 0 | M | no | no |  |  | no |  |
| 227 | 40 | II | M | no | no |  |  | Chl |  |

**Validation cohort**

| No. | Age | Rai-stage | IGHV mutation | 17p or 11q (%)* | Mutation TP53 or ATM (%)** | Amino acid change | Nucleotide change | Earlier treatment |
| --- | --- | --- | --- | --- | --- | --- | --- | --- |
| F1 | NA | NA | NA | 17p (83) | TP53 (15+25+60) | H214R  L252_I255del  R273H | 641A>G  754_762del(9bp)  818G>A | Chl, FCR, Al |
| 77 | 66 | IV | M | 17p (91) | TP53 (83) | R195* | 586C>T | no |
| 39 | 61 | III | M | 17p (82) | TP53 (85) | R273H | 818G>A | no |
| C2 | NA | NA | NA | 17p (95) | TP53 (100) | Q371X | 949C>T | no |
| 56 | 59 | III | NA | 17p (35) | TP53 (90) | C135G | 403T>C | no |
| G2 | NA | NA | NA | 17p (94) | TP53 (85) | E180_D181delinsR | 540_542delGCG | NA |
| 112 | 60 | IV | UM | no | TP53 (91) | M246V | 736A>G | no |
| 158 | 47 | IV | UM | no | TP53 (61) | C141fs | 420_429del(10bp) | no |
| 180 | 60 | III | UM | no | TP53 (94) | M246V | 736A>G | no |
| 239 | 72 | III | M | 17p (96) | no |  |  | no |
| 210 | 35 | I | UM | 17p (15) | no |  |  | no |
| 634 | 79 | I | NA | 17p (80) | no |  |  | NA |
| 653 | 61 | II | NA | 17p (50) | no |  |  | no |
| 188 | 49 | II | UM | 11q (32) | ATM | A1958E | 5873C>A | no |
| C1 | 65 | III | NA | 11q (98) | ATM | L2427P | 7280T>C | FC |
| 232 | 62 | III | NA | 11q (55) | ATM | V2367fs | 7101delT | no |
| 281 |  |  | NA | 11q (15) | ATM |  |  | no |
| 743 | 52 | IV | UM | 11q (80) | ATM | R2973W | 8917A>T | Chl, FCR, Al |
| 1075 | 73 | III | NA | 11q (85) | ATM | R2263K | 48_6788G>A | Chl, R-CVP, FCR, R-CHOP |
| B316 | 68 | III | UM | 11q (93) | ATM | F1025L | 3075T>G | no |
| B1075 | 68 | I | UM | 11q (98) | ATM | skipp.ex50 | 7089+1del | no |
| ATM MUT | NA | NA | NA | 11q (NA) | ATM | NA | NA | NA |
| 204 | 64 | IV | M | no | ATM | R13C | 37C>T | no |
| 27 | 56 | I | UM | no | ATM | D1753fs | 5203_5204insA | no |
| 11 | 43 | II | UM | no | ATM | N/A | 364delAATTAA | no |
| 719 | 68 | IV | UM | no | ATM | Y729STOP  E2423K | 2187C>G  7267G>A | F, R-CVP |
| B358 | 63 | III | UM | no | ATM | R3008C | 9022C>T | no |
| B75 | 80 | II | M | no | ATM | R3008H | 9023G>A | no |
| 107 | 68 | IV | NA | 11q (77) | no |  |  | no |
| 70 | 66 | IV | M | 11q (29) | no |  |  | no |
| 196 | 49 | I | UM | 11q (35) | no |  |  | no |
| 270 | 62 | IV | UM | 11q (18) | no |  |  | no |
| 52 | 60 | I | UM | 11q (94) | no |  |  | no |
| 109 | 45 | II | UM | 11q (46) | no |  |  | no |
| 34 | 38 | III | UM | 11q (17) | no |  |  | no |
| 164 | 46 | I | UM | 11q (15) | no |  |  | no |
| 785 | 59 | II | NA | 11q (95) | no |  |  | Chl, R, Of |
| 825 | 71 | III | NA | 11q (88) | no |  |  | NA |
| 1023 | 80 | II | NA | 11q (48) | no |  |  | Chl |
| 1072 | 27 | I | NA | 11q (41) | no |  |  | no |
| 6 | 63 | III | UM | no | no |  |  | no |
| 13_H | 60 | III | UM | no | no |  |  | no |
| 17 | 70 | III | UM | no | no |  |  | no |
| 19 | 56 | IV | UM | no | no |  |  | no |
| 20 | 57 | III | UM | no | no |  |  | no |
| 62 | 55 | III | M | no | no |  |  | no |
| 75 | 67 | I | UM | no | no |  |  | no |
| 83_H | 52 | I | UM | no | no |  |  | no |
| 91 | 61 | I | UM | no | no |  |  | no |
| 102 | 64 | II | UM | no | no |  |  | no |
| 152 | 49 | I | UM | no | no |  |  | no |
| 165 | 71 | II | UM | no | no |  |  | no |
| 182 | 69 | I | UM | no | no |  |  | no |
| 190 | 58 | IV | M | no | no |  |  | no |
| 194 | 47 | II | UM | no | no |  |  | no |
| 197 | 42 | |V | UM | no | no |  |  | no |
| 201 | 66 | IV | M | no | no |  |  | no |
| 212 | 46 | I | UM | no | no |  |  | no |
| 213 | 56 | IV | M | no | no |  |  | no |
| 218 | 75 | III | UM | no | no |  |  | no |
| 226 | 69 | III | NA | no | no |  |  | no |
| 230 | 63 | III | M | no | no |  |  | no |
| 261 | 59 | II | NA | no | no |  |  | no |
| 262 | 75 | IV | UM | no | no |  |  | no |
| B479 | 72 | 0 | UM | no | no |  |  | FCR |
| 717 | 73 | I | UM | no | no |  |  | no |
| 914 | 67 | IV | UM | no | no |  |  | Chl, FCR  R-CHOP |

Age and Rai-stage were determined at time of performance of RT-MLPA. FISH and mutational analysis were performed at same time point in most patients, but at least within one year. *% of mutant allele burden is mentioned from FISH if available, **% of mutant allele burden is mentioned from sequencing if available, ***Chemorefractory= not responsive towards either chlorambucil (Chl) or Fludarabine (F) is mentioned. Abbreviations: UPD=uniparental disomy, fs=frame shift, y=years, Chl=chlorambucil, FCR=fludarabine, cyclophosphamide and rituximab, Al=alemtuzumab, R-CHOP=rituximab, cyclophosphamide, doxorubicin, vincristine and prednisone, R-CVP= rituximab, cyclophosphamide, vincristin and prednisone, R-DHAP= rituximab, dexamethason, cytarabine and cisplatin, Of=Ofatumumab, NA=not available.

**Supplemental Table 2. Schematic overview of behavior of clusters of genes in either *TP53*/*ATM* WT, *TP53-*mutant or *ATM*-mutant CLL samples.**

|  | Cluster I | Cluster II | Cluster III | Cluster IV |
| --- | --- | --- | --- | --- |
| WT | ↑ | ↑ | ↑/↓ | ↓ |
| *TP53* MUT | **-** | ↑ | ↑ | ↓ |
| *ATM* MUT | - | - | - | - |

Table adapted from Stankovic et al^1^. Cluster I genes discriminate *TP53*/*ATM* WT from *TP53* and *ATM* mutated CLL cases. Cluster II-IV genes discriminate *TP53* from *ATM* mutated samples. ↑ = upregulation following irradiation, - = impaired upregulation or no upregulation following irradiation, ↓ = downregulation following irradiation, ↑/↓ = upregulation or downregulation following irradiation.

**Supplemental Table 3. Gene-specific oligonucleotides used for p53/ATM RT-MLPA probe set.**

| **HUGO name** | **Cluster** | **Probe** | **Length PCR** | **Chr. Pos** | **Genbank** | **Alias** | **Hybridising sequence of the small probe oligoncleotide** | **Spanning oligo** | **Hybridising sequence of the long probe oligonucleotide** | **Reverse Transcriptase primer sequence** |
| --- | --- | --- | --- | --- | --- | --- | --- | --- | --- | --- |
| **FAS** | I | 1 | 257 | 10q23.31 | NM_000043.4 | CD95, ALPS1A, APO-1, APT1, FAS1, FASTM, TNFRSF6 | CCTTCCAAATGCAGAAGATGTAGATTGTGTGATGAAGGACATGGCT |  | TAGAAGTGGAAATAAACTGCACCCGGACCCAGAATACC | AAAGTTTGGTTTACATCTGC |
| **FAS** | I | 2 | 328 | 10q23.31 | NM_000043.3 | CD95, ALPS1A, APO-1, APT1, FAS1, FASTM, TNFRSF6 | CACACTCACCAGCAACACCAAGTGCAAAGAGGAAG |  | GATCCAGATCTAACTTGGGGTGGCTTTGT | TGGCAAAAGAAGAAGACA |
| **BAX** | I | 1 | 214 | 19q13.33 | NM_138764.4 | BCL2L4 | GAAGATCCAAGACCAGGGTGGTTGGG |  | ACGGCCTCCTCTCCTACTTTGGGACGCCCACGTGG | ACGGTCTGCCACGTG |
| **BAX** | I | 2 | 220 | 19q13.33 | NM_138761.3 | BCL2L4 | CAGTAACATGGAGCTGCAGAGGA |  | TGATTGCCGCCGTGGACACAGACTCCCCCCGA | TCGGAAAAAGACCTCTCG |
| **BBC3** | I | 1 | 142 | 19q13.32 | NM_001127240.1 | JFY-1; JFY1; PUMA | TAAGAAGCCAGGAGAGGGACGGCTGAT |  | GGACTCAGCATCGGAAGGTGGCGGTGAC | CACCCCCTCGGTCA |
| **BBC3** | I | 2 | 364 | 19q13.32 | NM_001127240.2 | JFY-1; JFY1; PUMA | CTGCACAGTACGAGCGGCGGAGACAA |  | GAGGAGCAGCAGCGGCACCGCCCCTCACCCT | GATTGTACAGGACCCTCCA |
| **CDKN1A** | I | 1 | 130 | 06p21.2 | NM_078467.2 | P21; WAF1; CIP1; MDA6 | TCGGCAGACCAGCATGACAGAT |  | TTCTACCACTCCAAACGCCGGCTGATCTTCTCCAAGA | GGATTAGGGCTTCCTCTT |
| **CDKN1A** | I | 2 | 166 | 06p21.3 | NM_078467.2 | P21; WAF1; CIP1; MDA6 | CTTTGGACAGCAGAGGAAGACCATGTGGAC |  | CTGTCACTGTCTTGTACCCTTGTGCCTCGCTCAA | GGACCCTTCAGCCTGCT |
| **PCNA** | I | 1 | 244 | 20p13 | NM_002592.2 |  | GAACCCTTGTTGTAGAGTATAAAATTGCGGATATGGGACACTTAAAA |  | TACTACTTGGCTCCCAAGATCGAGGATGAAGAAGGATCTTAGGC | TCTTGAATTTTAAGAATGCCTAAGAT |
| **PCNA** | I | 2 | 250 | 20p13 | NM_002592.2 |  | GAAAGGATACCTTGGCGCTAGTATTTGAAGCACCAAAC |  | CAGGAGAAAGTTTCAGACTATGAAATGAAGTTGATGGATTTAGATGTTGAACAACTTGG | AGCTGTACTCCTGTTCTGGA |
| **PCNA** | I | 3 | 346 | 20p13 | NM_002592.2 |  | CTAGGAGAACTTGGAAATGGAAACATTAAATTGTCACAGACAAGTAA | TGTCGATAAAGAGGAGGAAGCTGTTACCATAGAGATGAATGAACCAGTTCAA | CTAACTTTTGCACTGAGGTACCTGAACTTCTTTACAAAAGCCACTCCAC | ACCGTTGAAGAGAGTGGAGT |
| **FDXR** | I | 1 | 178 | 17q25.1 | NM_024417.2 | ADXR | TAAGCCTAGCAGTCACTAGACTGGAGGGTG |  | TCGATGAGGCCACCCGTGCAGTGCCCAC | GAGGTCTTCCATGTCTCC |
| **FDXR** | I | 2 | 281 | 17q25.1 | NM_024417.2 | ADXR | GAAATGATCACCCCGAGGTGAAGAATGTCATCA |  | ACACATTTACCCAGACGGCCCATTCTGGCCG | CACAGCGGCCAGAAT |
| **NME1** | II | 1 | 196 | 17q21.33 | NM_198175.1 | AWD; GAAD; NB; NBS; NDKA; NDPK-A; NDPKA; NM23; NM23-H1 | TCCGTGGAGACTTCTGCATACAAGTTGGCAGGAACATTATA |  | CATGGCAGTGATTCTGTGGAGAGTGCAGA | AAGCCGATCTCCTTCTC |
| **NME1** | II | 2 | 265 | 17q21.33 | NM_198175.1 | AWD; GAAD; NB; NBS; NDKA; NDPK-A; NDPKA; NM23; NM23-H1 | GGATTCCGCCTTGTTGGTCTGAAATTCATGCA |  | AGCTTCCGAAGATCTTCTCAAGGAACACTACGTTGACCTGA | CAAAGAATGGACGGTCCT |
| **TNFRSF4** | II | 1 | 136 | 01p36.33 | NM_003327.3 | ACT35; CD134; IMD16; OX40; TXGP1L | TAAACGACCGGTGCTGCCACGAGTGCA |  | GGCCAGGCAACGGGATGGTGAGCCGCTGCAA | CACACCGTGTTCTGGGA |
| **TNFRSF4** | II | 2 | 319 | 01p36.33 | NM_003327.3 | ACT35; CD134; IMD16; OX40; TXGP1L | CAGTGCAAGCCCTGCACGTGGTGTAA |  | CCTCAGAAGTGGGAGTGAGCGGAAGCAGCTGT | GCAGACTGTGTCCTGTGT |
| **MYC** | III | 1 | 311 | 08q24.21 | NM_002467.4 | bHLHe39; c-Myc; MRTL; MYCC | CACCACCAGCAGCGACTCTGAGGA |  | GGAACAAGAAGATGAGGAAGAAATCGATGTTGTTTCTG | CCAGGAGCCTGCCTCTTTT |
| **MYC** | III | 2 | 337 | 08q24.21 | NM_002467.4 | bHLHe39; c-Myc; MRTL; MYCC | CAAACTTGCAGCTGCTTAGACGCTGGATTTTTTTCGGGTAGTGGAAAA |  | CCAGCAGCCTCCCGCGACGATGCCCCTC | GGTGAAGCTAACGTTGAG |
| **PYCR** | III | 1 | 238 | 17q25.3 | NM_153824.1 | ARCL2B; ARCL3B; P5C; P5CR; PIG45; PP222; PRO3; | GACTTCACAGCAGCAGGCGTCTTGGCT |  | GCCCACAAGATAATGGCTAGCTCCCCAGACATGA | TGGCCAGGTCCATGTCT |
| **PYCR** | III | 2 | 274 | 17q25.3 | NM_153824.1 | ARCL2B; ARCL3B; P5C; P5CR; PIG45; PP222; PRO3; | GAAACCATCAGCTCCATTGAGAAGAAGCTGTCA |  | GCGTTTCGGCCAGCCCCCAGGGTCATCCGCTGCATGA | CCACGACTGGAGTGTTG |
| **ASNS** | III | 1 | 154 | 07q21.3 | NM_133436.3 | ASNSD; TS11 | GTTTAAAGCAATGACAGAAGATGGATTTTTGGCTGTATGTTCAGAAGCTAAAGG |  | TCTTGTTACATTGAAGCACTCCGCGACTCCCTTT | GGAAGAAAAGGCTCCACTT |
| **ASNS** | III | 2 | 232 | 07q21.3 | NM_133436.3 | ASNSD; TS11 | CTTGGAAACTTATGACATTACAACAGTTCGTGCTTCAGTAGGTATGTATTTAATTTCCA |  | AGTATATTCGGAAGAACACAGATAGCGTGGTGATCTTCTCTGGAGAAGGA | CCTGCGTAAGTTCATCTG |
| **ACSM3** | IV | 1 | 202 | 16p12.2 | NM_005622.3 | SA; SAH | GTGTTCAGCACCAACTGTATACCGAATGCTTGTACA |  | GAATGATATAACCAGCTATAAGTTTAAAAGCTTAAAGCACTGTGTGAGTGCTGGGGAACCA | CGTCAGGGGTAATTGGTTC |
| **ACSM3** | IV | 2 | 301 | 16p12.3 | NM_005622.3 | SA; SAH | CTAAGAAACAAGTCTGCCATAATCTCCATCTGTGTTGGAATCTGT |  | TAACTAATGAACTGGTCTCTGTGCAAATCCTGAGTGCTAAAGCTTCCAACAA | CTAGCATCAGTCTTGTTGGAA |
| **ACSM3** | IV | 3 | 355 | 16p12.2 | NM_005622.3 | SA; SAH | CCTGGACCAATGGACTGATAAGGAAAAGGCTGGAAAGAA |  | ACCTTCAAATCCAGCCTTCTGGTGGATCAACAGAAATGGA | CGCATCTCTTCTCCATTTCTG |
| **AIFM1** | HK gene | 1 | 172 | Xq25 | NM_004208.3 | AIF; CMT2D; CMTX4; COWCK; COXPD6; NADMR; NAMSD; PDCD8 | CCCGAGGCAGAGGAACCGGCTCCCAG |  | GCAACTTGTTCCAGCGATGGCATGTTCCTCTAGAACTC | TTGTCTTGTCATCTGGAGTTC |
| **DIABLO** | HK gene | 1 | 124 | 12q24.31 | NM_019887.4 | DFNA64; SMAC | TCGCTTGAGCAGGTGATCATAGGAGCCAGAGCTGAGAT |  | GACTTCAAAACACCAAGAGTACTTGAAGCTGGAAACCACTTGGA | ACCAACTGCAGTCATCCA |
| **GUSB** | HK gene | 1 | 226 | 07q11.21 | NM_000181.3 | BG; MPS7 | CTATGCAGCAGACAAGGGGGCTCC |  | GTATGTGGATGTGATCTGTTTGAACAGCTACTACTCTTGGTAT | AGGTGCCCGTAGTCGT |
| **PARN** | HK gene | 1 | 373 | 16p13.1 | NM_002582.3 | DAN | CACTCCAGAAGAGAGGTATCAGAAGCTT |  | AAAAAGCATTCCATGGACTTTTTGCTATTTCAGTTTGGC | CATACTTAAAAGTGCAAAGGC |

**Supplemental Table 4. Overview of probes selected for further analysis.**

| Gene | Probe | P-value | FI_1_; geometric mean  FI WT | FI_2_; Geometric mean  FI *TP53* & *ATM* | FI_1_/FI_2_ |
| --- | --- | --- | --- | --- | --- |
| Cluster I | | | | | |
| ***FAS*** | **1** | **<0.0001** | 9.315 | 1.479 | **5.3** |
|  | 2 | <0.0001 | 8.830 | 1.865 | 4.7 |
| ***Bax*** | **1** | **<0.0001** | 4.395 | 1.917 | **2.4** |
|  | 2 | <0.0001 | 4.075 | 1.655 | 2.2 |
| ***BBC3*** | **1** | **<0.0001** | 6.781 | 1.955 | **3.2** |
|  | 2 | 0.01 | 1.900 | 1.178 | 1.6 |
| ***CDKN1A*** | **1** | **<0.0001** | 13.86 | 1.756 | **7.9** |
|  | 2 | <0.0001 | 4.459 | 1.350 | 3.3 |
| ***PCNA*** | **1** | **<0.0001** | 4.961 | 1.557 | **3.2** |
|  | 2 | <0.0001 | 4.011 | 1.493 | 2.7 |
|  | 3 | <0.0001 | 3.110 | 1.339 | 2.3 |
| ***FDXR*** | 1 | <0.0001 | 19.74 | 4.233 | 4.6 |
|  | **2** | **<0.0001** | 15.68 | 2.230 | **7.0** |
| Gene | Probe | P-value | FI_1_; geometric mean  FI *TP53* | FI_2_; geometric mean  FI *ATM* | FI_1_/FI_2_ |
| Cluster II | | | | | |
| ***NME1*** | **1** | **0.02** | 2.0 | 1.089 | **1.8** |
|  | 2 | 0.09 | 1.774 | 1.129 | 1.6 |
| *TNFRSF4* | 1 | 0.59 | 1.193 | 1.349 | 0.9 |
|  | 2 | 1.0 | 1.355 | 1.426 | 1.0 |
| Cluster III | | | | | |
| ***MYC*** | 1 | 0.22 | 0.8321 | 0.5915 | 1.4 |
|  | **2** | **0.02** | 0.9553 | 0.6134 | **1.6** |
| ***PYCR1*** | 1 | 0.14 | 1.284 | 0.7653 | 1.7 |
|  | **2** | **0.005** | 1.991 | 0.7836 | **2.5** |
| *ASNS* | 1 | 0.42 | 0.9258 | 0.8488 | 1.1 |
|  | 2 | 0.87 | 0.9681 | 0.9399 | 1.0 |
| Cluster IV | | | | | |
| ***ACSM3*** | 1 | 0.68 | 0.7423 | 0.8259 | 0.9 |
|  | **2** | **0.01** | 0.4997 | 0.9078 | **0.6** |
|  | 3 | 0.04 | 0.6473 | 1.123 | 0.6 |

For each selected gene, we selected the probe with the lowest p-value for the comparison of fold induction (FI) between (i) WT and *TP53*/*ATM* mutated CLL samples for cluster I genes and (ii) between *TP53*- and *ATM*-mutated CLL samples for cluster II-IV genes. In case of identical p-values, the probe with the largest change in FI between the two respective groups ( FI_1_/FI_2_), was selected. Probes selected for further analysis are highlighted in bold. Norminal p-values (Mann Whitney U test) and geometric means of fold inductions (FI) are indicated. FI_1_/FI_2_=geometric mean of FI of WT samples/ geometric mean of FI of *TP53*- /*ATM*-mutated samples for cluster I genes and geometric mean of FI of *TP53*-mutated/ geometric mean of FI of *ATM*-mutated samples for cluster II-IV genes.

**Supplemental Table 5. Reproducibility of individual genes in newly designed RT-MLPA.**

| **gene** | **geometric mean** | **95% CI** |
| --- | --- | --- |
| *FAS* | 8.1 | 7.7-8.6 |
| *Bax* | 4.3 | 4.1-4.5 |
| *BBC3* | 5.8 | 5.4-6.3 |
| *CDKN1A* | 14.4 | 13.5-15.5 |
| *PCNA* | 4.5 | 4.3-4.8 |
| *FDXR* | 14.0 | 11.8-16.7 |
| *NME1* | 1.84 | 1.75-1.92 |
| *MYC* | 0.69 | 0.64-0.74 |
| *PYCR1* | 1.03 | 0.90-1.19 |
| *ACSM3* | 0.41 | 0.37-0.46 |

Shown is an overview of the results of a sample which was performed in 23 times independent experiments in a period time of 3 years. Depicted are geometric mean of fold induction (FI) with 95% confidence interval (CI) for each individual gene of 23 independent experiments.

Reference List

1. Stankovic T, Hubank M, Cronin D et al. Microarray analysis reveals that TP53- and ATM-mutant B-CLLs share a defect in activating proapoptotic responses after DNA damage but are distinghuished by major differences in activating prosurvival responses. Blood 2004;103:291-300.
